# A Contribution to the Treatment of Epistaxis

**Published:** 1875-07

**Authors:** Beverly Robinson

**Affiliations:** Surgeon to the Manhattan Eye and Ear Hospital (Department of the Throat), etc.


### A CONTRIBUTION TO THE TREATMENT OF EPISTAPIS.

BY BEVERLEY ROBINSON, M.D.,

*Surgeon to the Manhattan Eye and Ear Hospital (Department of the Throat), etc.*

Epistaxis is no unfrequent trouble. At times it may be of slight importance, either because its efficient cause is ephemeral in nature, or else the loss of blood from the nasal passages is rather a fortunate circumstance than one to be regretted.

When, however, the patient is weak and laboring under some general diathetic dyscrasia, or when the epistaxis becomes abundant and the cause of it is obscure, we shall then have a condition of things which almost of necessity occasions solicitude.

Our desire and duty become in an equal degree imperative to to employ surely and rapidly such means as we may be able to utilize in order to arrest bleeding.

Generally speaking, recourse is had to cold, topically applied, and the employment of some one of the various mineral or vegetable astringents.

Simultaneously, or at a later period, drugs having a special corroborant or stypic action are prescribed, to be taken by the mouth.

To these have been added, within a brief period, those medicaments which act through the vaso-motor nerves, and tend to contract vascular walls. If all the above means fail of success, posterior, or both posterior and anterior plugging of the nasal passages, is considered and frequently put into execution.

What the trouble to the physician is, how great must be the annoyance and unpleasantness to the patient who submits to the operation we all know, either from our own experience or that of others. Besides, we are aware that it is only by care in the timely removal or renewal of the plugs that we can surely avoid certain attendant risks of septicæmia. Any mode of treatment, therefore, which has been adopted, when the agents usually employed have proved to be inefficacious, and has been productive of the happiest resnlts, will, we doubt not, receive a welcome from our colleagues.

Such a case, where obstinate bleeding was completely arrested by compression of the facial arteries, we shall now relate.

Case.—R. S., aged 62, medium constitution, residing in Savannah, Ga., came to New York city, and was received as an in-patient at the Manhattan Eye and Ear Hospital, September 4th, 1875, to be treated for cataract. A few days later, a successful operation was performed by Dr. Cornelius R. Agnew upon the affected eye, and during the first week of November the patient had recovered from the effects of the operation, and had obtained permission to return home. While out walking, November 11th, our patient was suddenly attacked with bleeding from the nose. The loss of blood at this time was quite abundant, and from November 11th to November 20th, notwithstanding the use of ordinary agents to arrest bleeding, the epistaxis frequently recurred during day and night. Finally the condition of the patent was such as to occasion much anxiety, both to herself and her medical attendants. Local applications of the astringent sort had been freely used, and these (alum, tannin, etc.) were insufflated, under the form of powder, into the nasal passages, and also employed in strong solution, by means of the posterior nasal syringe. Cold compresses upon the nose and forehead were also tried, aud the patient was placed in a room at a somewhat lowered temperature.

By the application of the topics above mentioned, clots were formed in the nasal passages, and the bleeding stopped temporarily.

In a few hours, however, oozing of blood took place about the margin of the anterior clots (especially the one on the left side) and between them and the alæ of the nose.

Two days after this mere local treatment was begun, large doses of quinine, given internally, were added to it. And this was done for two reasons:

1st. Because the patient informed us that she had had a severe attack of malarial fever six years previously, and since that time occasionally experienced chilly sensations, which she attributed to this poisoning.

2d. We believe that, from the well known action of sulphate of quinine to contract or lessen the calibre of small vessels, we might fairly hope it would be of service.

Unfortunately we were deceived in our anticipations, and though we gave as much as thirty grains of quinine in eighteen hours, and continued it during three days, it remained without appreciable effect. We were prevented from still further increasing the dose by very unpleasant symptoms of giddiness and buzzing in the ears.

On November 16th large doses of tincture of the chloride of iron (30 gtt.), combined with small doses of quinine (2 gr.) were prescribed to be taken every four hours; also similar doses of fluid extract of ergot (viz., 30 gtt.) every four hours, with the injunction that the ergot should be given two hours before or after the exhibition of the iron and quinine. In this way the patient would not be more than two hours, except during those of sleep, without taking medicine internally.

The styptic and tonic action of the iron, as well as the influence of ergot on the vaso-motor nerves, and through them on muscular fibre, remained equally inefficacious. After two or three days of the above treatment, the clots were washed from the nasal passages, and plugging by means of the India-rubber bag, as recommended by Salvatore Caro, M.D. (vide *Med. Record*, August 15th, 1874), was attempted. This method proved equally fruitless with what had already been tried in stopping the flow of blood.

We now commenced, whilst continuing the use of iron, quinine and ergot as above, compression of the facial arteries upon the superior maxillary bones, just before they reach the alæ of the nose, by means of two small pads made of lint. These were sewed to a piece of tape at the proper distance from one another, and the ends of the tape were passed across the cheeks and above the ears, and tied securely behind the occipatal bone.

This treatment achieved the happiest consequences, as will be seen from the following notes:

November 23d. Patient continues to take fluid extract of ergot and tincture of iron, with small doses of quinine. Compression of facial arteries almost unceasing since first applied. On one occasion only the tape and pads were removed in order to ease the patient, who complained somewhat of the discomfort caused by them, and the bleeding immediately recommenced. General condition improved; patient stronger and less anxious.

*Treatment.*—Continue iron and quinine, and pressure with pads on facial arteries; nourishing food; discontinue ergot.

November 24th. Patient continues to improve in strength and morale. So soon, however, as she takes pressure off facial arteries, bleeding reoccurs.

November 30th. Bleeding has stopped altogether for several days. Patient left hospital.

We do not, of course, believe that by compression of the facial arteries we shall be able to arrest all cases of bleeding from the nose. We trust, however, it may frequently be adjoined to other treatment with marked benefit to our patient, and by itself may prove of the greatest utility in exceptional cases where other means are not at hand. For, in point of fact, it is from the septum that takes origin many of our worst cases of epistaxis, and it is this portion of the nasal passages which receives its arteries mainly from the terminal branches of the facial. By compression of the arterial trunk we must, of course, greatly diminish, if we do not stop absolutely, the afferent blood flow.

This treatment, then, is *rational*, and whenever experience is capped with reason we are better satisfied with, and more willing to accept it.

A word with respect to the instrument described and lauded by Dr. Salvatore Caro, and to which we made reference in the history of our case. We regret to say that in our hands the instrument did not work satisfactorily, and for the following reasons:

1. When *air* is blown in by the tube the bag dilates unequally, and, besides, dilates either more or less than we desire. And, further, we have no means of accurately determining how much the bag has in reality dilated, except our tactile sensations, which are furnished by the right index finger passed behind the velum. Of course if the inflated bag should come into contact with the pharynx, where we are able to see it, *gagging* is, of necessity, immediately occasioned.

2. The sides of the catheter attached to the bag are too *firm*, and when we used pressure on them with the fingers, or my means of a cord tightly tied, so as to prevent the return of the air blown into he bag, we found it impossible to accomplish our object.

3. Owing to its unequal dilatation, the bag is apt to give way in some particular point, anb allow the air to escape, and consequently the sides of the bag to collapse. Are these objections avoidable? We believe they are, if the following indications be carried out:

1. The bag should be made of rubber, which shall dilate *equally* and the amount of dilation to be given may be limited by an outer covering to the bag, which is inelastic and sufficiently strong to resist a certain amount of interior pressure.

This outer bag should have a capacity, when expanded, somewhat superior to the dimensions of the posterior nares, and be made of some very light, smooth material.

2. The conductor-tube should have a faucet attached to it near its distal extremity, so that the air or water could be shut up in the bag as soon as it is filled with either one of these fluids.*

